# Association of weight-adjusted waist index and Albuminuria in children and adolescents: A national population-based study

**DOI:** 10.1371/journal.pone.0324354

**Published:** 2025-07-23

**Authors:** Jiawen Huo, Jianfeng Liu, Jiying Chen, Qiaolin Li, lanling Shen, Juanjuan Liang, Jie Jiang

**Affiliations:** 1 Department of Paediatrics, The Affiliated Second Hospital, Hengyang Medical school, University of South China, Hengyang, China; 2 Department of Nephrology, The Affiliated Second Hospital, Hengyang Medical school, University of South China, Hengyang, China; Sri Ramachandra Institute of Higher Education and Research (Deemed to be University), INDIA

## Abstract

**Background:**

Albuminuria is a recognized marker of early kidney damage and cardiometabolic risk in pediatric populations. While central obesity is known to contribute to renal dysfunction, the relevance of the weight-adjusted waist index (WWI), a novel indicator of central adiposity, has not been fully explored in children and adolescents.

**Methods:**

This study included 4,000 participants aged 3–19 years from the National Health and Nutrition Examination Survey (NHANES) 2017–2020. WWI was calculated as waist circumference divided by the square root of body weight. Albuminuria was defined as an albumin-creatinine ratio (ACR) > 30 mg/g. Multivariable logistic regression, subgroup analyses, threshold effect modeling, and receiver operating characteristic (ROC) curves were used to evaluate the association between WWI and albuminuria.

**Results:**

Higher WWI was significantly associated with lower odds of albuminuria in the fully adjusted model (OR = 0.64; 95% CI: 0.55–0.75). This inverse relationship was strongest among adolescents (13–19 years), modest in children aged 7–12 years, and not significant in the 3–6-year group. In the youngest group, a U-shaped association was identified, with an inflection point at 11.73 cm/√kg. ROC analysis showed WWI had superior discriminatory ability (AUC = 0.628) for albuminuria compared to BMI, waist circumference, height, and weight.

**Conclusion:**

WWI demonstrates an age-dependent and non-linear association with albuminuria in U.S. children and adolescents. These findings suggest that WWI may offer a more refined anthropometric indicator of renal risk in youth and support its potential as a screening tool in pediatric populations.

## 1. Introduction

Albuminuria, characterized by the presence of albumin in urine, is a well-recognized marker of kidney damage and is predictive of both chronic kidney disease (CKD) and cardiovascular morbidity and mortality in adults [1,2]. In pediatric populations, the presence of albuminuria is of particular concern, as it may indicate early renal damage, which could progress to more severe conditions if left untreated [3]. Moreover, albuminuria in children has been associated with other metabolic and cardiovascular risk factors, suggesting that it may serve as an early indicator of broader health risks [4].

Obesity, particularly central obesity, is a major public health issue in both adults and children. Central obesity has been linked to a range of adverse health outcomes, including insulin resistance, hypertension, dyslipidemia, and CKD [5,6]. Traditional measures of obesity, such as body mass index (BMI), do not fully capture the distribution of body fat, particularly the visceral fat that is most closely associated with metabolic risk [7]. The weight-adjusted waist index (WWI), which adjusts waist circumference by the square root of body weight, has been proposed as a more sensitive measure of central obesity [8]. Unlike BMI, WWI specifically accounts for abdominal fat distribution, making it potentially more relevant to conditions like albuminuria [9,10].

Despite the known associations between central obesity and renal outcomes in adults, the relationship between WWI and albuminuria in children and adolescents remains poorly understood. Understanding this relationship is crucial for early intervention, as it could help identify at-risk individuals who may benefit from targeted prevention strategies. This study aims to investigate the association between WWI and albuminuria in a nationally representative sample of U.S. children and adolescents, using data from the National Health and Nutrition Examination Survey (NHANES) 2017–2020.

## 2. Methods

This study utilized data from the NHANES 2017–2020, a program designed to assess the health and nutritional status of adults and children in the United States through interviews, physical examinations, and laboratory tests [11]. NHANES employs a complex, multistage probability sampling design to ensure that the survey results are representative of the U.S. civilian non-institutionalized population. Participants aged 3–19 years were included in the analysis.

**Fig 1:**
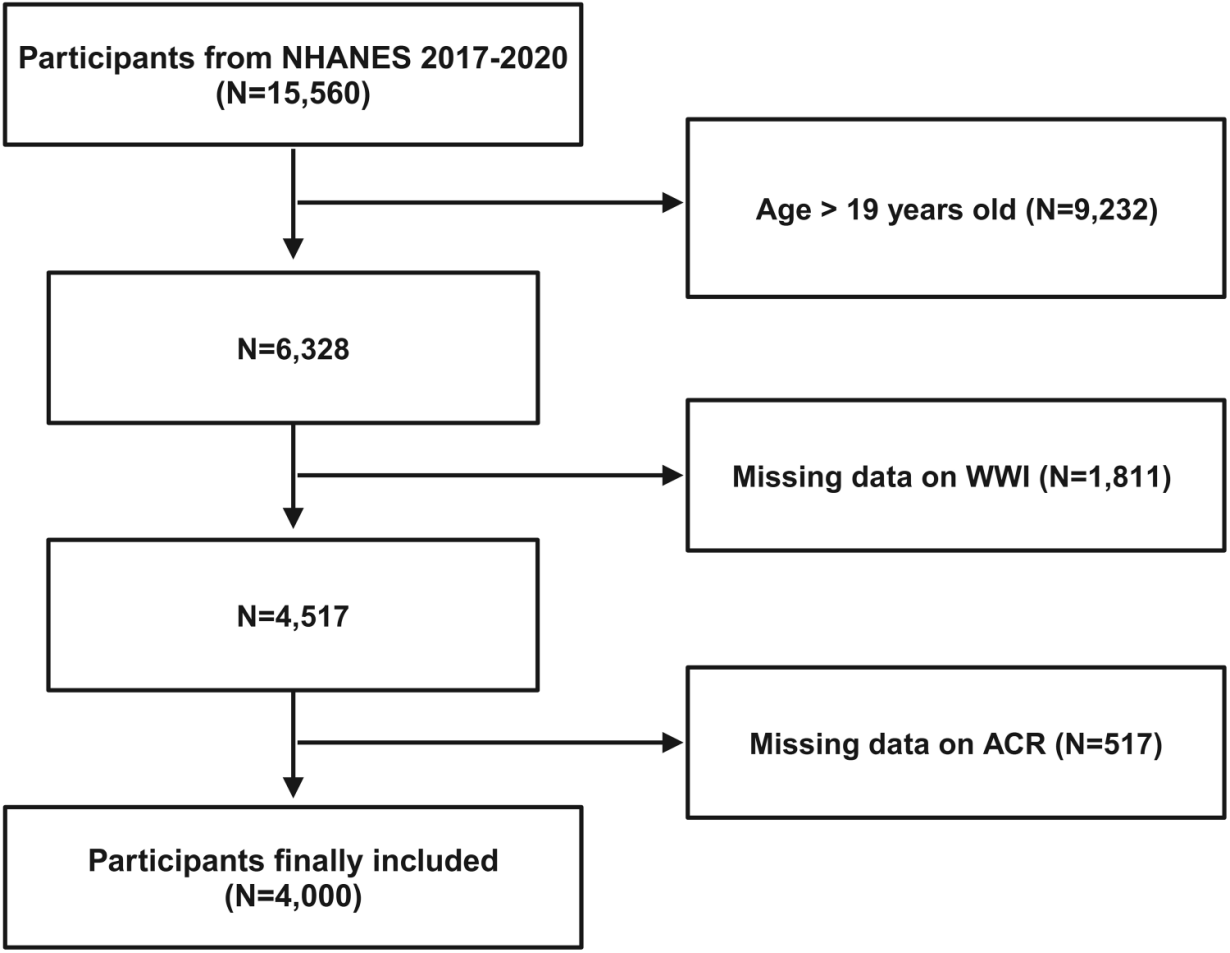
Flow chart of participant’s selection. NHANES, National Health and Nutrition Examination Survey.

From an initial pool of 15,560 participants, individuals with age older than 19 years old were excluded (N = 9,232). In addition, participants without valid measurements for waist circumference or body weight (N = 1,811), or those lacking urine albumin and creatinine data (N = 517), were excluded from the analysis, resulting in a final analytic sample of 4,000 children and adolescents (Fig 1).

### Weight-Adjusted Waist Index (WWI)

The primary exposure, weight-adjusted waist index (WWI), was calculated as waist circumference (in centimeters) divided by the square root of body weight (in kilograms) [12]. This index was chosen because it adjusts for overall body size while focusing on central adiposity, which may be more relevant for assessing metabolic risk in pediatric populations. WWI has been proposed as a measure that adjusts for body size and more precisely captures central adiposity, potentially offering advantages over BMI or waist circumference alone in pediatric metabolic studies [13].

### Albuminuria

The outcome variable was albuminuria, defined using the albumin-creatinine ratio (ACR), calculated from urinary albumin (mg) divided by urinary creatinine (g). In accordance with established clinical thresholds, albuminuria was defined as ACR > 30 mg/g [14].

### Covariates

Covariates included in the analysis were age (categorized into three groups: 3–6 years, 7–12 years, 13–19 years), sex, race/ethnicity (Non-Hispanic White, Non-Hispanic Black, Mexican American, and Other race/multiracial), diabetes status, total cholesterol, HDL-C, BMI, and the ratio of family income to poverty (PIR).

### Statistical analysis

All statistical analyses were conducted using R version 4.3.0 and empowerstat version 4.2, with appropriate survey weights to account for the complex sampling design of NHANES. Missing variables were imputed using random forest–based multiple imputation via the “missForest” package in R (version 1.5.0) [15,16].

Multivariate logistic regression models were employed to estimate the odds ratios (ORs) for albuminuria associated with WWI. Three models were constructed: Model 1 included no covariates, Model 2 adjusted for age, sex, and race/ethnicity, and Model 3 further adjusted for diabetes status, BMI, total cholesterol, HDL-C, and PIR. The analysis primarily focused on WWI as a continuous variable, and additional analyses explored its association with albuminuria across different quartiles of WWI to assess potential non-linear relationships. To explore potential non-linear relationships between WWI and albuminuria, smooth curve fitting techniques were employed. These techniques allow for the flexible modeling of associations between continuous variables without assuming a strictly linear relationship. This approach was particularly useful for identifying inflection points and characterizing the relationship between WWI and albuminuria across the distribution of WWI. Subgroup analyses were conducted to assess whether the association between WWI and albuminuria varied by age, sex, and race/ethnicity. Interaction terms were included in the logistic regression models to formally test for effect modification by these variables, allowing for a more nuanced understanding of how demographic factors might influence the relationship between WWI and albuminuria. In children aged 3–6 years, a threshold effect analysis was performed to identify a potential inflection point in the relationship between WWI and albuminuria [17]. In addition, ROC curves comparing the discriminatory ability of WWI, BMI, waist circumference, height, and weight for predicting albuminuria were constructed [18].

## 3. Results

### Baseline characteristics

The final analytic sample consisted of 4,000 children and adolescents, with a mean age of 10.87 years (SD ± 4.64). Of these participants, 50.1% were male, and 49.9% were female. The cohort included 485 (12.12%) children or adolescents who were diagnosed with albuminuria. The mean WWI in the total population was 11.47 cm/√kg (SD = 1.24), with a range from 7.91 to 14.80 cm/√kg. Participants were categorized into quartiles based on their WWI, with significant differences observed across these quartiles in terms of age, sex, race/ethnicity, BMI, PIR, total cholesterol, and HDL-C levels (Table 1).

**Table 1:** Basic characteristics of participants by weight-adjusted waist index quartile.

Characteristics	Q1 (N = 1000)	Q2 (N = 1000)	Q3 (N = 1000)	Q4 (N = 1000)	*P*-value
Age, n (%)					<0.001
3-6 years	0 (0.00%)	27 (2.70%)	203 (20.30%)	632 (63.20%)	
7-12 years	206 (20.60%)	500 (50.00%)	594 (59.40%)	312 (31.20%)	
13-19 years	794 (79.40%)	473 (47.30%)	203 (20.30%)	56 (5.60%)	
Sex, n (%)					<0.001
Male	612 (61.20%)	463 (46.30%)	491 (49.10%)	491 (49.10%)	
Female	388 (38.80%)	537 (53.70%)	509 (50.90%)	509 (50.90%)	
Race/ethnicity, n (%)					<0.001
Non-Hispanic White	297 (29.70%)	285 (28.50%)	300 (30.00%)	356 (35.60%)	
Non-Hispanic Black	363 (36.30%)	267 (26.70%)	245 (24.50%)	188 (18.80%)	
Mexican American	95 (9.50%)	152 (15.20%)	181 (18.10%)	182 (18.20%)	
Other race/multiracial	245 (24.50%)	296 (29.60%)	274 (27.40%)	274 (27.40%)	
Diabetes, n (%)					0.022
Yes	6 (0.60%)	16 (1.60%)	12 (1.20%)	4 (0.40%)	
No	994 (99.40%)	984 (98.40%)	988 (98.80%)	996 (99.60%)	
Albuminuria, n (%)					0.550
Yes	119 (11.90%)	129 (12.90%)	110 (11.00%)	127 (12.70%)	
No	881 (88.10%)	871 (87.10%)	890 (89.00%)	873 (87.30%)	
BMI (kg/m2)	21.43 ± 4.00	22.27 ± 6.51	21.39 ± 7.37	19.83 ± 6.83	<0.001
PIR	2.24 ± 1.61	2.19 ± 1.63	2.11 ± 1.58	1.99 ± 1.55	0.004
HDL-C (mg/dL)	54.52 ± 11.94	53.64 ± 12.00	53.45 ± 12.19	49.01 ± 11.01	<0.001
Total cholesterol (mg/dL)	151.06 ± 27.43	154.64 ± 28.96	159.53 ± 28.75	159.17 ± 27.54	<0.001
Albumin-creatinine ratio (mg/g)	22.90 ± 72.81	38.70 ± 266.27	23.97 ± 78.15	22.04 ± 111.86	<0.001

Mean ± SD for continuous variables: the P value was calculated by the weighted linear regression model.

(%) for categorical variables: the P value was calculated by the weighted chi-square test.

Abbreviation: Q, quartile; PIR, Ratio of family income to poverty; BMI, body mass index; HDL-C, High-density lipoprotein cholesterol.

Participants in the highest WWI quartile were more likely to be older, female, and Non-Hispanic White. They also had higher mean total cholesterol levels and lower HDL-C levels. Interestingly, the prevalence of diabetes was lower in the highest WWI quartile compared to the lowest quartile (0.4% vs. 0.6%), though this difference was not statistically significant.

### Association between WWI and Albuminuria

**Fig 2:**
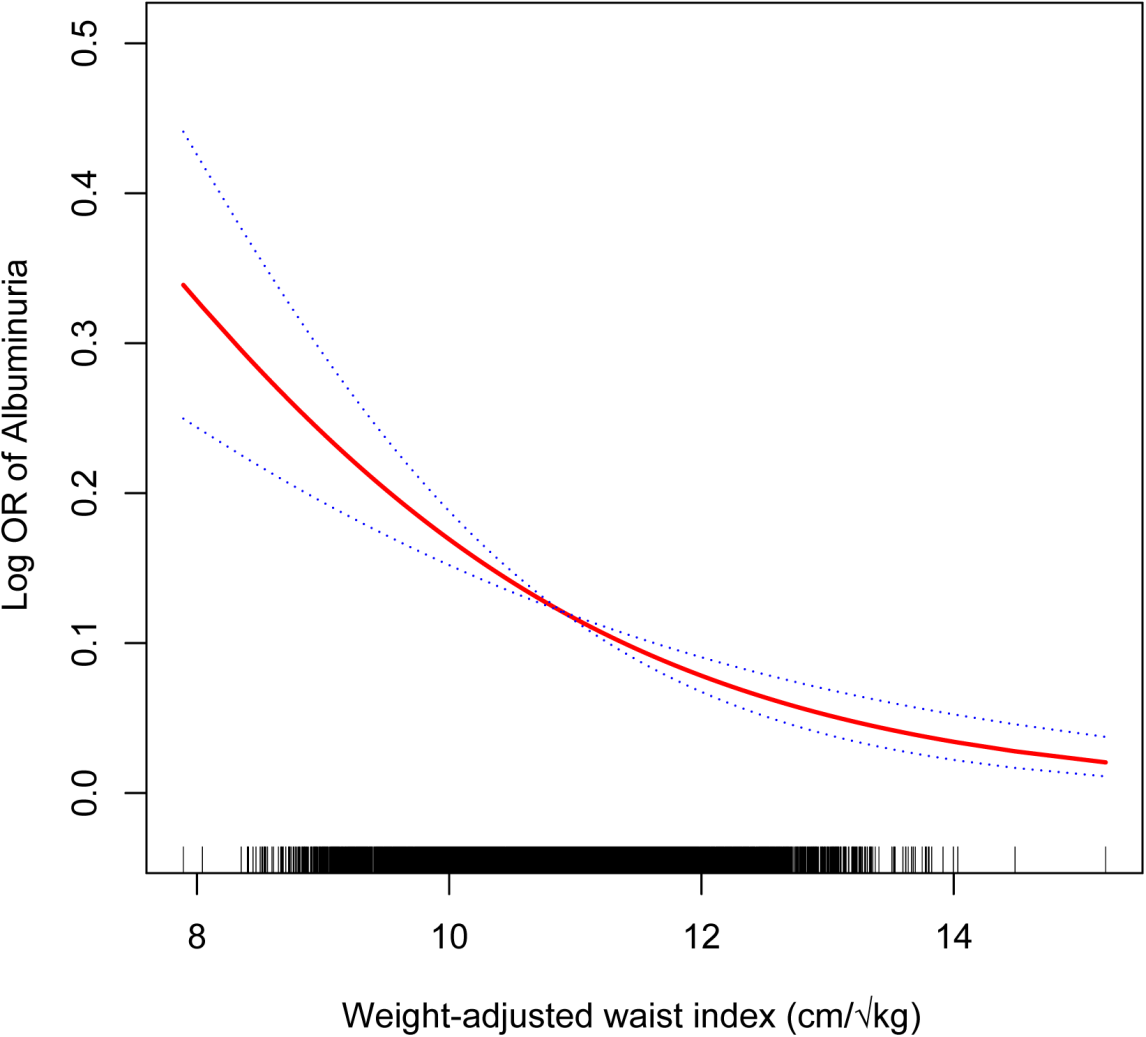
The nonlinear associations between weight-adjusted waist index and albuminuria. The solid red line represents the smooth curve fit between variables. Blue bands represent the 95% of confidence interval from the fit.

Table 2 presents the associations between WWI and albuminuria. In the unadjusted model (Model 1), WWI was not significantly associated with albuminuria (OR=1.00, 95% CI: 0.92–1.09). However, after adjusting for age, sex, and race/ethnicity (Model 2), a significant inverse association was observed (OR=0.65, 95% CI: 0.56–0.75). This association remained significant in the fully adjusted model (Model 3), where each unit increase in WWI was associated with a 36% reduction in the odds of albuminuria (OR=0.64, 95% CI: 0.55–0.75). When participants were categorized into WWI quartiles, those in the highest quartile had a 62% lower odds of albuminuria compared to those in the lowest quartile (OR=0.38, 95% CI: 0.25–0.59). A significant linear trend was observed across quartiles (P for trend < 0.001), suggesting that higher WWI is consistently associated with lower odds of albuminuria (Table 2). Fig 2 further confirmed the inverse association between WWI and albuminuria in children and adolescents.

**Table 2:** The associations between weight-adjusted waist index and albuminuria.

WWI	Model 1 [OR (95% CI)]	Model 2 [OR (95% CI)]	Model 3 [OR (95% CI)]
**Continous WWI (cm/√kg)**	1.00 (0.92, 1.09)	0.65 (0.56, 0.75)	0.64 (0.55, 0.75)
**Quartiles**			
Quartile 1	1 (ref)	1 (ref)	1 (ref)
Quartile 2	1.10 (0.84, 1.43)	0.76 (0.57, 1.01)	0.81 (0.60, 1.09)
Quartile 3	0.92 (0.69, 1.21)	0.47 (0.34, 0.65)	0.46 (0.32, 0.66)
Quartile 4	1.08 (0.82, 1.41)	0.39 (0.26, 0.58)	0.38 (0.25, 0.59)
P for trend	0.869	<0.001	<0.001

Model 1: no covariates were adjusted. Model 2: age, sex, and race were adjusted. Model 3: age, sex, race, diabetes status, total cholesterol, HDL-C and PIR were adjusted. Abbreviation: PIR, Ratio of family income to poverty; HDL-C, High-density lipoprotein cholesterol.

**Fig 3:**
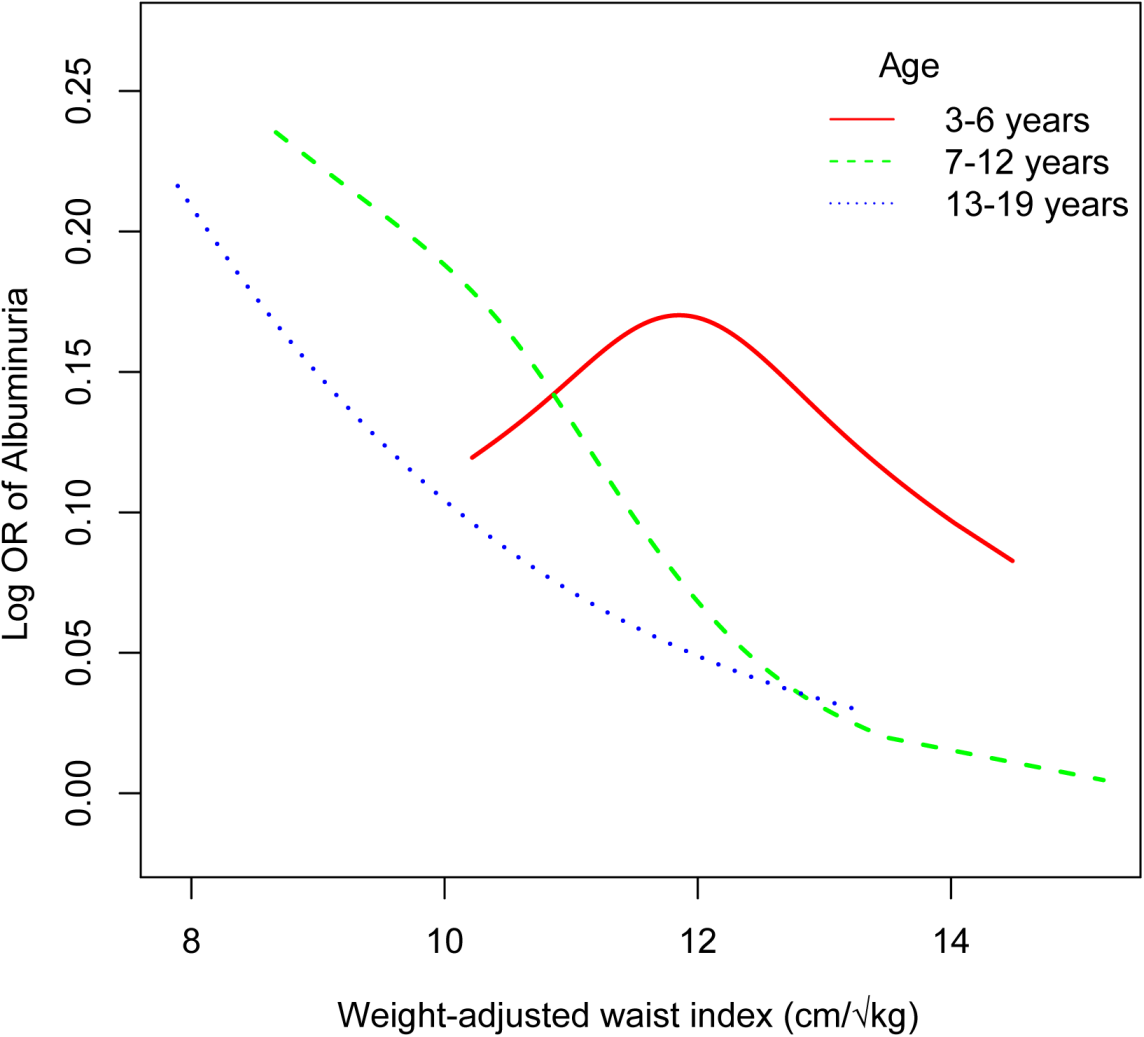
Receiver operating characteristic (ROC) curves comparing the predictive performance of WWI, body mass index (BMI), waist circumference (WC), height, and weight for albuminuria.

The ROC analysis (Fig 3) comparing anthropometric predictors of albuminuria showed that WWI had the highest discriminatory power (AUC = 0.628), followed by BMI (0.587), waist circumference (0.605), height (0.555), and weight (0.572).

### Subgroup analysis

Subgroup analysis (Table 3) revealed that the inverse relationship between WWI and albuminuria was strongest in adolescents aged 13–19 years (OR = 0.67; 95% CI: 0.52–0.86), followed by children aged 7–12 years (OR = 0.75; 95% CI: 0.60–0.94), and was weakest and non-significant in children aged 3–6 years (OR = 0.91; 95% CI: 0.67–1.24). These findings are now discussed in greater detail. An interaction by age group was significant (P for interaction = 0.029), while sex and race/ethnicity did not significantly modify the association (P > 0.05).

**Table 3:** Subgroup analysis of the association between weight-adjusted waist index and albuminuria.

Subgroup	Albuminuria [OR (95%CI)]	P for interaction
Sex		0.052
Male	0.54 (0.42, 0.68)	
Female	0.71 (0.60, 0.85)	
Age		0.029
3-6 years	0.93 (0.71, 1.24)	
7-12 years	0.58 (0.47, 0.72)	
13-19 years	0.67 (0.52, 0.86)	
Race/ethnicity		0.096
Non-Hispanic White	0.51 (0.39, 0.66)	
Non-Hispanic Black	0.72 (0.55, 0.95)	
Mexican American	0.58 (0.41, 0.82)	
Other race/multiracial	0.80 (0.60, 1.05)	

### Threshold effect analysis

**Fig 4:**
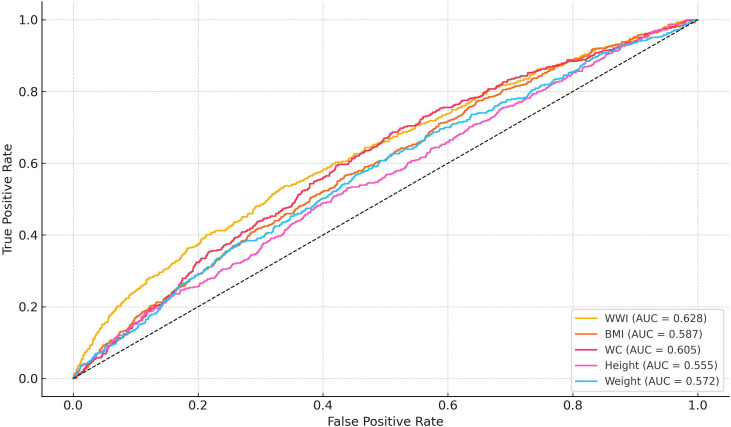
The nonlinear associations between weight-adjusted waist index and albuminuria by different age groups.

In children aged 3–6 years, the analysis identified a U-shaped association between WWI and albuminuria, with a significant inflection point at a WWI of 11.73 cm/√kg (Table 4, Fig 4). Below this threshold, increases in WWI were associated with higher odds of albuminuria, indicating a potential risk associated with lower levels of central obesity. However, above this threshold, the relationship reversed, with higher WWI associated with lower odds of albuminuria, suggesting a complex interplay between body composition and renal function in this young age group.

**Table 4:** Threshold effect analysis of weight-adjusted waist index on albuminuria by two-piecewise linear regression model.

WWI (cm/√kg)	Fitting by the standard linear model	Fitting by the two-piecewise linear model			
Inflection point (K)	< K-segment effect	> K-segment effect	Log likelihood ratio
3-6 years participants	0.93 (0.71, 1.24)	11.73	2.26 (0.88, 5.80)	0.69 (0.45, 1.04)	0.040

Models were adjusted for age, sex, race, diabetes status, total cholesterol, HDL-C and PIR were adjusted. Abbreviation: PIR, Ratio of family income to poverty; HDL-C, High-density lipoprotein cholesterol.

## 4. Discussion

This study provides novel insights into the association between the WWI and albuminuria among U.S. children and adolescents. Contrary to the initial hypothesis that higher WWI, indicative of central obesity, would be associated with increased odds of albuminuria, our findings suggest an inverse relationship, particularly in older children and adolescents. Specifically, adolescents aged 13–19 exhibited the strongest inverse relationship, while children aged 7–12 showed a similar but slightly weaker inverse association. In contrast, in the youngest age group (3–6 years), a U-shaped association was observed, with a significant inflection point at a WWI of 11.73 cm/√kg, indicating that the relationship between central obesity and albuminuria may be more complex and influenced by distinct developmental and metabolic processes.

### Comparison with previous studies

The association between obesity and albuminuria has been extensively studied in adults, where central obesity is consistently associated with an increased risk of albuminuria and subsequent CKD [19–21]. However, studies focusing on children and adolescents present a more nuanced picture, often revealing age-dependent and obesity-type specific associations that differ from those observed in adults [22].

Several pediatric studies have found that higher BMI, a common measure of overall obesity, is associated with an increased risk of albuminuria [3,23–27]. For example, a large cross-sectional study using data from the NHANES reported that obese children had significantly higher odds of albuminuria compared to their normal-weight peers [28]. Similarly, research by Burgert et al. demonstrated that obese adolescents exhibited higher levels of albuminuria, which were closely associated with markers of insulin resistance [29].

However, BMI does not differentiate between fat distribution types, particularly central versus peripheral adiposity [30]. Central obesity, characterized by excessive visceral fat, is more closely linked to metabolic abnormalities and adverse renal outcomes [31]. Studies that specifically focus on central obesity, such as waist circumference or waist-to-height ratio, have shown stronger associations with albuminuria in children [32,33]. For example, a study by Rodríguez et al. reported that waist circumference was a better predictor of albuminuria than BMI in Spanish children, underscoring the importance of considering fat distribution in assessing renal risk [34]. Our ROC curve analysis further substantiates WWI’s superior predictive capability for albuminuria (AUC: WWI = 0.628, BMI = 0.587, WC = 0.605, height = 0.555, weight = 0.572).

One plausible explanation for this unexpected finding is that WWI potentially captures a favorable body composition profile involving a higher proportion of lean muscle mass relative to fat mass. During adolescence, physiological changes such as increased muscle accrual can positively impact insulin sensitivity, reduce systemic inflammation, and mitigate renal stress [35–38]. Supporting this hypothesis, studies demonstrate that higher lean mass correlates with reduced insulin resistance and improved kidney function, possibly due to enhanced glucose uptake, reduced adipokine dysregulation, and diminished oxidative stress [39,40].

Furthermore, the U-shaped association observed in younger children (3–6 years) adds complexity, suggesting differential physiological mechanisms at extreme WWI values. Higher WWI might reflect early-stage metabolic disruptions causing renal microvascular stress, endothelial dysfunction, and consequent albumin leakage [41,42]. Conversely, the increased albuminuria risk at low WWI values may reflect nutritional deficiencies or inadequate adipose reserves vital for renal and metabolic development. Undernutrition in early childhood adversely affects nephron endowment and renal functional reserve, elevating susceptibility to renal damage and proteinuria [43–45].

The divergence of our findings from previous studies underscores the necessity of age-specific interpretations and highlights how dynamic changes in body composition during growth could influence renal outcomes [46–48]. Future studies using more refined and direct body composition measures, such as dual-energy X-ray absorptiometry (DEXA), are essential to validate and explore these hypotheses rigorously.

### Potential biological mechanisms

Several biological mechanisms could underlie the observed associations. In adolescents, a higher WWI may represent a protective body composition characterized by greater muscle mass and relatively lower visceral fat accumulation. Increased muscle mass can enhance metabolic efficiency, reduce insulin resistance, and decrease systemic inflammation, all of which are recognized contributors to renal injury and albuminuria [49,50]. Muscle-derived myokines, such as irisin and IL-15, could further exert renoprotective effects by modulating renal inflammation, oxidative stress, and fibrosis [51]. These pathways represent plausible biological underpinnings for the inverse WWI-albuminuria relationship in adolescents.

In younger children (3–6 years), the observed U-shaped relationship likely reflects complex interactions between nutritional status, adiposity distribution, and renal developmental physiology. At low WWI levels, inadequate fat stores and altered growth trajectories could negatively influence nephrogenesis, renal functional reserve, and vascular development, thereby increasing susceptibility to albuminuria through impaired endothelial integrity or glomerular hypertrophy [52,53]. At higher WWI values, increased visceral fat may trigger early insulin resistance, endothelial dysfunction, and elevated pro-inflammatory cytokines (e.g., TNF-α, IL-6), exacerbating renal microvascular injury and protein leakage [54,55]. Threshold analysis supports this intricate relationship, emphasizing the necessity of developmental context when interpreting WWI’s impact on renal health.

### Clinical implications

From a clinical perspective, while WWI is not yet established as a diagnostic criterion for pediatric renal dysfunction, its potential utility as an exploratory, non-invasive screening tool warrants attention. Routine monitoring of WWI in clinical practice may facilitate early identification of renal risk, particularly in adolescents, guiding targeted preventive lifestyle interventions (e.g., dietary optimization, physical activity enhancement) to mitigate future kidney disease risks [56,57]. Nonetheless, clinical implementation of WWI requires further validation through prospective longitudinal studies.

### Strengths and limitations

This study has several strengths, including the use of a large, nationally representative sample and the application of a novel obesity metric. The comprehensive adjustment for potential confounders enhances the validity of the findings, making them generalizable to the broader U.S. pediatric population.

However, limitations exist. The cross-sectional design precludes causal inference, emphasizing the need for longitudinal studies to ascertain temporality and causation definitively. Furthermore, our analysis lacked molecular and genetic data, precluding examination of underlying mechanistic pathways at the genetic or biochemical level, such as genes involved in calcium metabolism or bone mineral regulation that might influence renal outcomes. Future mechanistic studies integrating genetic and biochemical markers are needed to elucidate these pathways fully [58]. Moreover, since the majority of participants were classified as normal weight based on BMI, caution is warranted when generalizing the findings to pediatric populations with a higher prevalence of obesity. Finally, reliance on some self-reported covariates introduces potential reporting bias, which future studies should address through objective measurements.

## 5. Conclusion

This study highlights a nuanced and age-dependent relationship between WWI and albuminuria in U.S. children and adolescents. Contrary to expectations, higher WWI inversely associates with albuminuria in older children, whereas younger children demonstrate a U-shaped association. These findings underscore the complexity of body composition’s impact on renal health across different pediatric stages and suggest WWI’s potential as a valuable screening metric. Future longitudinal and mechanistic research is crucial to validate these associations and explore underlying biological processes thoroughly.

## Supporting information

S1 Data (XLS)

## Abbreviations

ACR: Albumin-creatinine ratio
BMI: body mass index
CKD: Chronic kidney disease
DXEA: Dual-energy X-ray absorptiometry
NHANES: National Health and Nutrition Examination Survey
NCHS: National Center for Health Statistics
PIR: Ratio of family income to poverty
WWI: Weight-adjusted waist index.
